# Natural Products Against Renal Fibrosis *via* Modulation of SUMOylation

**DOI:** 10.3389/fphar.2022.800810

**Published:** 2022-03-04

**Authors:** Peng Liu, Jing Zhang, Yun Wang, Chen Wang, Xinping Qiu, Dan-Qian Chen

**Affiliations:** ^1^ Shunyi Hospital, Beijing Hospital of Traditional Chinese Medicine, Beijing, China; ^2^ Institute of Plant Resources, Yunnan University, Kunming, China; ^3^ Department of Emergency, China-Japan Friendship Hospital, Beijing, China

**Keywords:** renal fibrosis, sumoylation, SUMO-specific protease, natural products, NF-κB

## Abstract

Renal fibrosis is the common and final pathological process of kidney diseases. As a dynamic and reversible post-translational modification, SUMOylation and deSUMOylation of transcriptional factors and key mediators significantly affect the development of renal fibrosis. Recent advances suggest that SUMOylation functions as the promising intervening target against renal fibrosis, and natural products prevent renal fibrosis *via* modulating SUMOylation. Here, we introduce the mechanism of SUMOylation in renal fibrosis and therapeutic effects of natural products. This process starts by summarizing the key mediators and enzymes during SUMOylation and deSUMOylation and its regulation role in transcriptional factors and key mediators in renal fibrosis, then linking the mechanism findings of SUMOylation and natural products to develop novel therapeutic candidates for treating renal fibrosis, and concludes by commenting on promising therapeutic targets and candidate natural products in renal fibrosis via modulating SUMOylation, which highlights modulating SUMOylation as a promising strategy for natural products against renal fibrosis.

## 1 Introduction

Epigenetic modification is dynamic and responses to environmental influences, including chromatin remodeling proteins and histone post-translational modifications (PTM). SUMOylation is a PTM process. SUMOylation shares similar reaction scheme and enzyme classes, but the conjugation involves in small ubiquitin-like modifiers (SUMOs) rather than ubiquitin. Emerging evidences support SUMOylation as a promising therapeutic target for renal fibrosis through modulating transcription factor and key mediator. NR5A2 is a key mediator on the transcriptional regulation of the calreticulin gene, and its SUMOylation exacerbates renal fibrosis in the unilateral ureteric obstruction model (Arvaniti et al., 2016). In addition, SUMOylation of Samd4 by SUMO2/3 activates TGF-β/Smad signaling and then upregulates the expression of fibronectin in high glucose induced renal mesangial cells (Zhou et al., 2014). Here, we introduce the mechanism of SUMOylation in renal fibrosis and therapeutic effects of natural products. This process starts by summarizing the key mediators and enzymes during SUMOylation and deSUMOylation and its regulation role in transcriptional factors and key mediators in renal fibrosis, then linking the mechanism findings of SUMOylation and natural products to develop novel therapeutic candidates for treating renal fibrosis, and concludes by commenting on promising therapeutic targets and candidate natural products in renal fibrosis via modulating SUMOylation.

## 2 SUMOylation

### 2.1 SUMO Proteins

**FIGURE 1:**
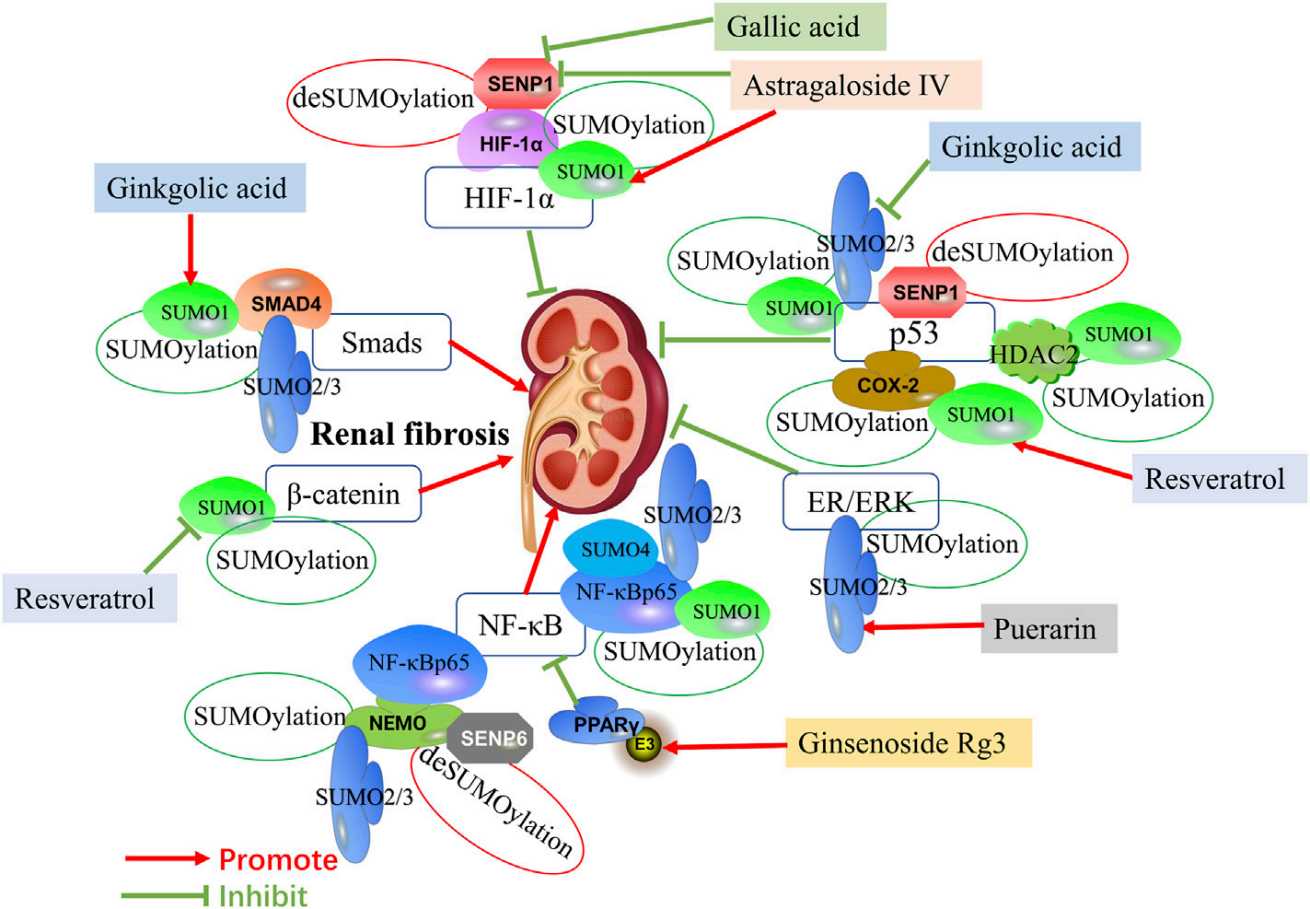
Mechanisms of SUMOylation and deSUMOylation by transcription factors in renal fibrosis and therapeutic targets of natural products against renal fibrosis *via* SUMOylation. Mechanisms of SUMOylation and deSUMOylation by transcription factors in renal fibrosis are including TGF-β/Smad signaling, HIF-1α signaling, p53 pathway, ER/ERK pathway, NF-κB signaling, and β-catenin pathway. Smad4 is SUMOylated by SUMO1 and SUMO2/3. HIF-1α is SUMOylated by SUMO1. P53 is SUMOylated by SUMO1 and SUMO2/3, and also is upregulate by HDAC2 and COX-2 SUMOylated by SUMO1. ER/ERK is SUMOylated by SUMO2/3. NF-κBp65 is SUMOylated by SUMO1, SUMO2/3 and SUMO4, and also is upregulate by NEMO SUMOylated by SUMO2/3. PPARγ activated by SUMO ligase E3 could inhibit NF-κB signaling. β-catenin is SUMOylated by SUMO1. SNEP1 deSUMOylates HIF-1α and p53. Additionally, SNEP6 deSUMOylates NEMO. Ginkgolic acid promotes the expression of Smad4 by upregulating SUMO1, while inhibits the expression of p53 by downregulating SUMO2/3. Astragaloside IV promotes the expression of HIF-1α by upregulating SUMO1 and downregulating SENP1. Gallic acid also promotes the expression of HIF-1α by downregulating SENP1. Resveratrol induces SUMOylated COX-2 by SUMO1, thus enhancing the expression of p53. In addition, Resveratrol inhibits the expression of β-catenin by downregulating SUMO1. Puerarin activates ER/ERK pathway by upregulating SUMO2/3. Ginsenoside Rg3 inhibits NF-κB by upregulating PPARγ via activating E3.

SUMO proteins are small acidic proteins with distant homology to ubiquitin. At primary amino acid sequence, they are 10–20% identical to ubiquitin. On a structural level, and their relatedness is much more pronounced. As shown in Figure 1, ubiquitin and SUMO share the classical ubiquitin superfold. Characteristic for all members of the SUMO family and absent from ubiquitin or other ubiquitin-related proteins, is the N-terminal flexible extension of 10–30 amino acids. The function of this extension remains however currently unknown.

As a type of PTMs, SUMOylation are widely existed in eukaryotes. SUMOs modification mediates the protein–protein interactions of target substrates, and affects their enzymatic function, subcellular localization, and stability (Kukkula et al., 2021). SUMO proteins covalently bind to their target proteins at lysine (k) residues. To date, five SUMO proteins (SUMO1–5) have been found in human genome harbors genes (Enserink, 2015). Due to the sequence of SUMO2 and SUMO3 is very similar, they are commonly known as SUMO2/3 (Saitoh and Hinchey, 2000). Although SUMO2/3 and SUMO1 share 50% sequence homology, significant differences have been shown in function and location. Interestingly, SUMO1 knockout mice are survivable because SUMO2/3 can compensate for most functions of SUMO1 (Zhang et al., 2008; Wang et al., 2014a). SUMO1 and SUMO2/3 are common, while SUMO4 is only expressed in few organs, such as spleen and kidney (Du et al., 2021). SUMO4 is associated with the pathogenesis of type I diabetes mellitus. SUMO5 is first discovered in promyelocytic leukemia, which mediates the growth and disruption of promyelocytic leukemia nuclear bodies (Liang et al., 2016). However, whether SUMO5 is expressed at the protein level remains controversial.

### 2.2 SUMOylation and deSUMOylation Process

The SUMOylation starts from precursor synthesis, hydrolytic activation to covalent binding to the substrate protein that relies on the synergistic effect of three enzymes: E1 (SUMO-activating enzyme); E2 (SUMO- transferring enzyme); and E3 (SUMO ligase) (Yeh, 2009). First, SUMOs are activated by E1, and the C-terminal glycine is connected to the cysteine residue of E1 through a thiolipid bond. Thus, its group is adenylated to provide ATP energy and completes the activation of SUMOs. Then, SUMOs are transferred to the cysteine residues of E2, which can directly recognize the substrate, and finally couple SUMO to the lysine of the substrate protein through an isopeptide bond residues. There are three main types of E3: activated STAT protein inhibits protein inhibitor of activated STAT (PIAS) family members, Pc2 and Ran BP2. It can enhance the efficiency and specificity of E2 transferring SUMOs to substrate proteins (K et al., 2021).

As a highly regulated and reversible process, SUMOylation is regulated by a series of proteases (Wilkinson and Henley, 2010). As one of the key proteases, SUMO-specific proteases (SENPs) contain seven types (SENP1–3 and SENP5–8). SENP1/2 prefers SUMO1–3 as the substrate (Wilkinson and Henley, 2010), while SENP3/5 and SENP6/7 have broad specificity for SUMO2/3 (Mukhopadhyay and Dasso, 2007), and SENP8 does not act on SUMO protein (Kunz et al., 2018). Under the action of SENPs, SUMOs are cleaved from the substrate protein, and the SUMOs precursor is processed into mature SUMOs re-enter the new SUMO cycle.

The abnormal SUMOylation usually leads to disorders of function of target proteins, and causes the damage of important physiological processes (Bertke et al., 2019). The balance of SUMOylation and deSUMOylation is crucial for embryonic development, since knockout of SENP1, SENP2, or SENP3 leads to embryonic lethality in mice (Chiu et al., 2008; Lao et al., 2018).

## 3 SUMOylation in Renal Fibrosis

SUMOs involve in many cellular processes including DNA damage repair, protein stability regulation, cell cycle progression and regulation of signal transduction (Eifler and Vertegaal, 2015; Zeng et al., 2020). Consequently, abnormal SUMO modification can also results in many diseases such as cancers, diabetes, liver diseases, and kidney diseases (Bettermann et al., 2012; Li et al., 2019; Yang et al., 2019; Xie et al., 2020; Zeng et al., 2020). Especially, SUMOylation is activated by some factors, such as hypoxia, metabolic stress, oxidation, genotoxicity, and osmosis (Sun et al., 2018; Appelman et al., 2021; Ma et al., 2021).

**FIGURE 2:**
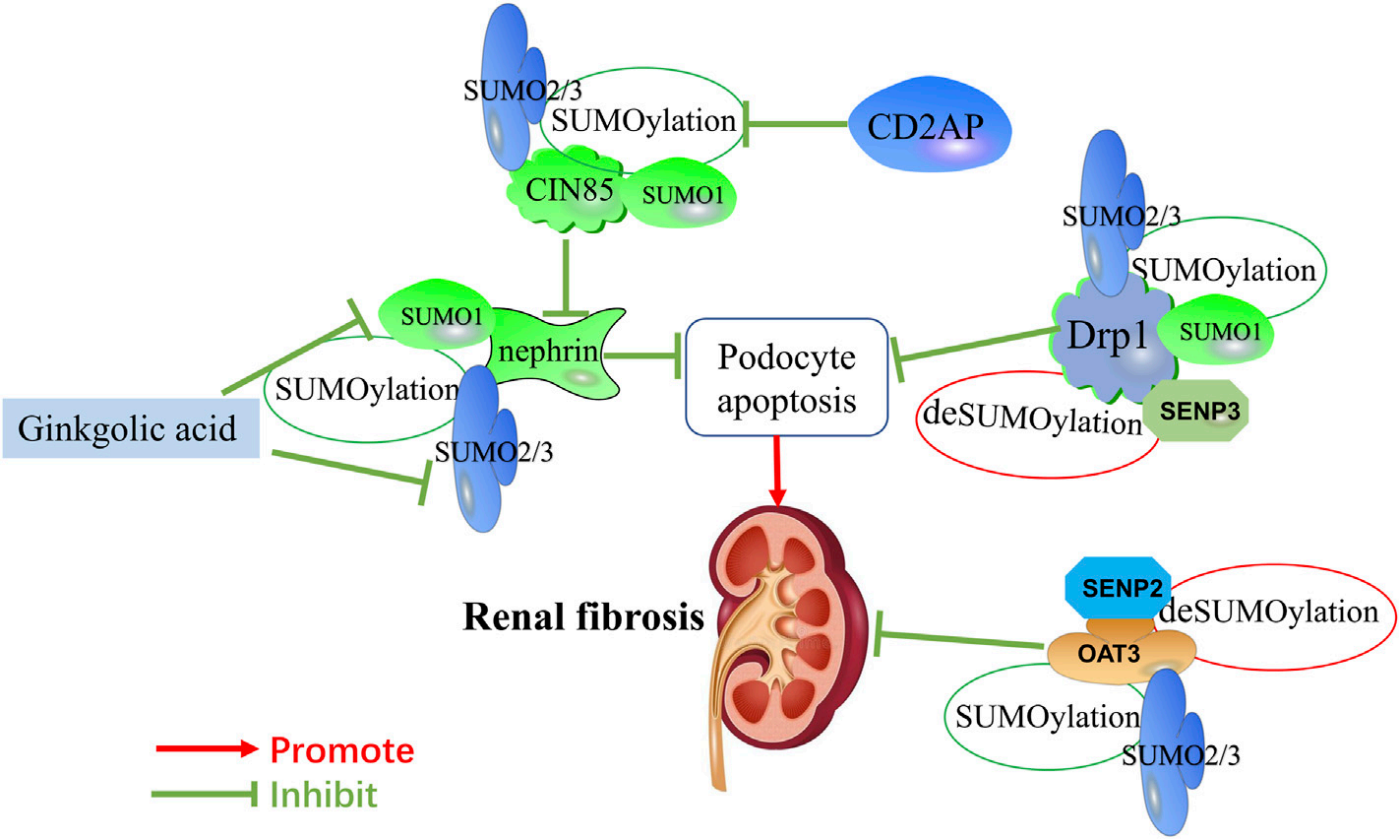
Mechanisms of SUMOylation and deSUMOylation by key mediators in renal fibrosis and therapeutic targets of natural products against renal fibrosis via SUMOylation. Mechanisms of SUMOylation and deSUMOylation by key mediators in renal fibrosis are including podocyte apoptosis, and OAT3-mediated transfer channels. As one of component of the slit diaphragm, nephrin is SUMOylated by SUMO1 and SUMO2/3. CD2AP promotes the expression of nephrin by inhibiting the SUMOylation of CIN85. Drp1 is SUMOylated by SUMO1 and SUMO2/3. OAT3 is SUMOylated by SUMO2/3. SNEP3 deSUMOylates Drp1, and SNEP2 deSUMOylates OAT3. Ginkgolic acid inhibits the expression of nephrin by downregulating SUMO1 and SUMO2/3.

Notably, recent studies indicate that SUMOylation functions as a critical role in maintaining renal fibrosis process. As the final pathological process of kidney diseases, renal fibrosis is caused by a decrease in matrix degradation, inflammatory cell infiltration, dysregulation of cell-matrix interaction, and the accumulation of extracellular matrix (ECM) proteins, which mainly involves fibronectin, various collagen and laminin. Although many researches have described the pathogenesis of renal fibrosis, few studies have investigated the role and mechanism of protein SUMOylation during renal fibrosis. Under certain pathological conditions, the signal pathway regulated by SUMOylation is associated with the pathophysiological changes of renal fibrosis. The regulation of renal fibrosis processes involves multiple signaling pathways, therefore we will elaborate on the SUMOylation of transcriptional factors and key mediators in renal fibrosis (Table 1). The molecular mechanisms involved in SUMOs and renal fibrosis are present in Figure 1 and Figure 2.

**TABLE 1:** The SUMOylation of transcription factors and key mediators in renal fibrosis.

Model	SUMO proteins or enzymes	Target genes	References
Modulating transcription factors			
High glucose induced renal mesangial cells	SUMO2/3	Smad4	Zhou et al. (2014)
Senp1(−/−) mice	SENP1	HIF-1α	Xu et al. (2010)
Co-culture models of glomerular endothelial cells with podocytes	SENP1	HIF-1α	Wang et al. (2015)
Puromycin aminonucleoside-induced podocyte	SENP1	p53	Wang et al. (2014)
Renal mesangial cells	SUMO1	p53	Wu et al. (2021)
Rat kidney proximal tubular cells	SUMO2/3	p53	Guo et al. (2015)
HEK 293T, HCT- 116 p53^−/−^ and all RKO cells	SUMO1	p53	Wagner et al. (2015)
High glucose stimulated rat glomerular mesangial cells	SUMO1 and SUMO2/3	NF-κB p65	Huang et al. (2013)
High glucose stimulated rat glomerular mesangial cells	E3	NF-κB p65	Huang et al. (2017)
Lipopolysaccharide (LPS)-induced human renal proximal tubular cells	E3	PPARγ	Lu et al. (2013)
diabetic GK rats	SUMO4	NF-κB p65	Chen et al. (2014)
C57BL/6 mice, HEK293T, MEF and RAW264.7 cells	SUMO2/3	NEMO/IKKγ	Liu et al. (2013)
C57BL/6 mice, HEK293T, MEF and RAW264.7 cells	SENP6	NEMO	Liu et al. (2013)
Modulating key mediators			
C57/Bl6 mice and podocytes HEK-293T	SUMO1 and SUMO2/3	Nephrin	Tossidou et al. (2014)
Podocytes	SUMO1 and SUMO2/3	CIN85	Tossidou et al. (2012)
HEK293 and HeLa cells	SENP3	Drp1	Guo et al. (2017)
COS-7 fibroblasts	SENP2	OAT3	Wang and You, (2019)
COS-7 fibroblasts	SUMO2/3	OAT3	Wang et al. (2019)

### 3.1 The SUMOylation of Transcription Factors in Renal Fibrosis

#### 3.1.1 Smads

TGF-β/Smad signaling pathways play a critical role in renal fibrosis through Smad and non-Smad pathways (Meng et al., 2015; Frangogiannis, 2020). Sumoylation of type I TGF-β receptor increases its ligand recruitment ability by phosphorylation of Smad3 (Kang et al., 2008). Smad3 is involved in TGF-β-mediated signaling pathway. E3 ligase PIASy suppresses TGF-β signaling through SUMOylating Smad3 and Smad4 (Imoto et al., 2008; Lee et al., 2014). SUMOylated Smad4 increases the activation of TGF-β1/Smad signaling (Lee et al., 2003; Long et al., 2004; Wang Z. et al., 2018). In high glucose induced renal mesangial cells, SUMOylation of Samd4 by SUMO2/3 activates TGF-β/Smad signaling and then upregulates the expression of fibronectin (Zhou et al., 2014). In addition, some studies reveal that SUMO1 also enhances Smad4 SUMOylation (Ohshima and Shimotohno, 2003; Wang Z. et al., 2018; Liu et al., 2020). Ski and SnoN can inhibit TGF-β pathway through blocking its interaction with Smads. Arkadia (a RING domain E3 ubiquitin ligase) mediates noncovalent interaction with poly-SUMO2, and activates TGF-β/Smad signaling via degradation of Ski and SnoN (Erker et al., 2013). Taken together, targeting SUMOylation of TGF-β/Smad signaling might function as a promising therapeutic approach against renal fibrosis.

#### 3.1.2 HIF-1α Pathways

Decreased oxygen significantly affects gene expression, metabolic changes and regeneration processes, including angiogenesis and stimulation of stem cell proliferation, differentiation, and migration (Hadanny and Efrati, 2020). Glomerular damage leads to a decrease in oxygen supply, and recent studies have emphasized the key role of hypoxia in the development of renal fibrosis (Bessho et al., 2019; Ishiuchi et al., 2020; Wakashima et al., 2020). The expression of SUMO proteins is increased under conditions of hypoxia *in vivo* and *in vitro* (Comerford et al., 2003; Shao et al., 2004).

The expression of hypoxia-inducible factor-1α (HIF-1α) in kidney is upregulated under conditions of hypoxia (Bessho et al., 2019). Meanwhile, the upregulation of HIF-1α expression can improve the viability and angiogenesis of bone marrow mesenchymal stem cells (Luo et al., 2019). Evidences show that vascular endothelial growth factor A (VEGFA) promotes the survival of bone marrow mesenchymal stem cells and angiogenesis, while HIF-1α can upregulate the expression of VEGFA (Utispan and Koontongkaew, 2021; Wang B. et al., 2021). Hypoxia enhances the SUMOylation of HIF-1α by SUMO1 and SUMO2/3 (Carbia-Nagashima et al., 2007); whereas SENP1 could deconjugate SUMOylated HIF-1α and reduce HIF-1α degradation during hypoxia (Cheng et al., 2007). SENP1 enhances HIF-1α deSUMOylation and increases VEGF production in endothelial cells following exposure to hypoxia (Xu et al., 2010; Cui et al., 2017). In SENP1(−/−) mice, the vascular endothelial cells in embryonic renal glomeruli and the VEGF production were significantly reduced (Xu et al., 2010). In co-culture models of glomerular endothelial cells with podocytes, the expression of SENP1 in podocytes increases under conditions of hypoxia, which induces endothelial cells survival via deSUMOylation of HIF-1α signaling (Wang et al., 2015). Therefore, SUMOylation of HIF-1α has the potential to be clinically developed as an antifibrotic target.

#### 3.1.3 p53 Pathways

Podocyte apoptosis is a main cause of the decrease in the number of podocytes, which can lead to proteinuria, glomerulosclerosis and renal fibrosis. Recent studies found that the p53 protein participates in the pathogenesis of podocyte apoptosis (Choy et al., 2021; Liang et al., 2021; Yang et al., 2021). As a tumor suppressor, p53 could safeguard the genome and prevent malignant transformation by halting the cell cycle and promoting apoptosis (Jiang et al., 2021; Thoms and Stark, 2021). p53 is increased under stimulation, and then upregulates the expression of pro-apoptotic genes (Eliaš and Macnamara, 2021). SUMOylated p53 could bind to the anti-apoptotic factor Bax and Bcl-2, and lead to apoptosis (Nowakowski et al., 2021). SENP1 deficiency significantly increases podocyte apoptosis by increasing expression of the p53 target pro-apoptotic genes (Noxa, PUMA and BAX) and aggravating accumulation of SUMOylated p53 protein in puromycin aminonucleoside-induced podocyte injury (Wang et al., 2014b). Mesangial cell proliferation is a core pathological feature of many kidney diseases. In renal mesangial cells, Krϋppel-like factor 15 (KLF15) targeted SUMO1 to inhibit cell proliferation via enhancing the stability of p53 (Wu et al., 2021). However, ginkgolic acid (a pharmacological inhibitor of SUMOylation) suppresses p53 SUMOylation by SUMO2/3 and enhances apoptosis in rat kidney proximal tubular cells (Guo et al., 2015). SUMOylation of histone deacetylase 2 (HDAC2) by SUMO1 reduced DNA damage-induced apoptosis via deacetylation of p53 (Wagner et al., 2015).

#### 3.1.4 NF-κB Signaling

As a nuclear transcription factor, nuclear factor κB (NF-κB) regulates the accumulation and release of cytokines and adhesion molecules by leukocytes (Wenzl et al., 2021). During the resting state, NF-κB binds to the inhibitor of κB (IκB) in the cytoplasm (Lee et al., 2021). SUMOylation of NF-κB plays a core role in the regulation of renal inflammation and fibrosis (Al Za’abi et al., 2021; You et al., 2021). With the modification by SUMO2/3, IκBα is separated from NF-κB, and enhance the activation of NF-κB (Chang and Abe, 2016). In high glucose stimulated rat glomerular mesangial cells, IκBα SUMOylation reduces the expression of monocyte chemotactic protein 1 (MCP-1), and ameliorates cellular inflammatory response through inhibiting NF-κB signaling (Huang et al., 2013). As one of E3 ligases, PIAS promotes SUMOylation of NF-κB via enhancing the expression of SUMO1 and SUMO2/3, and leads to the release of MCP-1 and IL-6 from glomerular mesangial cells (Huang et al., 2017).

The utilization of renal energy is partially determined by peroxisome proliferator activated receptors (PPARs) mediated fatty acid oxidation (Aranda-Rivera et al., 2021). PPARγ plays a key role in physiological and pathological processes of renal fibrosis. The reduction of PPARγ SUMOylation suppresses the activation of anti-apoptotic and anti-inflammatory cytokines through inhibiting the NF-κB activity (Xie et al., 2018; Zhao et al., 2021b). In lipopolysaccharide (LPS)-induced human renal proximal tubular cells, rosiglitazone (an agonist of PPARγ) decreases chemokines expression via inhibiting NF-κB activation by activating PPARγ SUMOylation (Lu et al., 2013). SUMO4 also plays a key role in regulating NF-κB signaling in glomerular cells (Chen et al., 2014). SUMOylation NEMO/IKKγ (NF-κB essential modifier) by SUMO2/3 enhanced the activity of NF-κB pathway, whereas SENP6 reverses this process by catalyzing the deSUMOylation of NEMO (Liu et al., 2013). These results indicate that the regulation of NF-κB SUMOylation has become a potential therapeutic strategy for renal fibrosis.

### 3.2 The SUMOylation of Key Mediators in Renal Fibrosis

#### 3.2.1 Podocyte Apoptosis Pathway

Podocyte injury is the basic pathological feature of many kidney diseases, including decreased expression of the fissure membrane components and ultrastructural changes. Recently, SUMOylation has also been implicated in podocyte injury. As one of components of the slit diaphragm, nephrin is a target protein for SUMOylation by SUMO1 and SUMO2/3 (Tossidou et al., 2014). In podocytes, SUMOylation plays a key role in the tight orchestration of nephrin turnover at the slit diaphragm. The SUMOylation inhibitor ginkgolic acid decreases PI3K/AKT signaling and reduces membrane expression of nephrin (Tossidou et al., 2014). Besides nephrin, CD2-associated protein (CD2AP) also plays a crucial role in slit diaphragm (Chung et al., 2020; Basgen et al., 2021). Due to high sequence and structural similarities with CD2AP, Cbl-interacting protein of 85 kDa (CIN85) is a binding partner of nephrin and then increases endocytosis of nephrin (Tossidou et al., 2010). CD2AP reduces the binding of CIN85 to nephrin via CIN85 SUMOylation (Tossidou et al., 2012).

Mitochondrial dysfunction caused excessive mitochondrial fission, and then promoted podocyte apoptosis through the overproduction of reactive oxygen species (Su et al., 2021; Zhu et al., 2021). Moreover, mitochondrial dysfunction in damaged podocytes causes the development of marked glomerulosclerosis and albuminuria (Zhou et al., 2019). SUMOylation of dynamin-related protein (Drp) 1 activates mitochondrial autophagy and inhibits ROS production *via* regulating mitochondrial morphology and division (Din et al., 2013). SUMO1 SUMOylates Drp1, and then prevents the mitochondrial translocation (Jin et al., 2021). On the other hand, SENP2 deSUMOylates Drp1 by removing SUMO1, whereas SENP3 and SENP5 removes SUMO2/3 from deSUMOylation of Drp1 (Fu et al., 2014; Shimizu et al., 2016). DeSUMOylation of Drp1 by SENP3 promotes cell death by enhancing Drp1 partitioning to the mitochondrial outer membrane and improving cytochrome c release and apoptosis (Guo et al., 2017).

#### 3.2.2 OAT3-Mediated Transfer Channels

Organic anion transporter 3 (OAT3) is an important transporter that located in the basolateral membrane of proximal renal tubules. OAT3 plays vital parts in removing a variety of drugs from the kidney, so as to avoid their possibly toxic side effects in the body (Fu et al., 2021). Activated OAT3 attenuates renal lipid accumulation and alleviates renal injury associated with renal inflammation and fibrosis in high-fat diet-induced obese rats (Pengrattanachot et al., 2020). In COS-7 fibroblasts, the downregulation of Senp2 results in an increased OAT3 SUMOylation via enhancing OAT3 expression and transport activity (Wang and You, 2019). In addition, protein kinase A (PKA) accelerates the OAT3 SUMOylation by SUMO2/3 (Wang et al., 2019).

## 4 Natural Products Against Renal Fibrosis via Regulating SUMOylation

### 4.1 Natural Products Against Renal Fibrosis via Regulating the SUMOylation of Transcription Factors

Numerous natural products have been widely applied to the treatment of fibrosis via inhibition of SUMOylation (Table 2). However, few studies have investigated the role and mechanism of natural products against renal fibrosis *via* modulating SUMOylation. Recent studies reveal that ginkgolic acid, a natural product from ginkgo, could regulate the SUMOylation of p53 to attenuate renal fibrosis (Tossidou et al., 2014; Guo et al., 2015).

**TABLE 2:** Natural products and therapeutic targets against renal fibrosis via SUMOylation.

Compounds	Resource	SUMO proteins or enzymes	Therapeutic target	References
Modulating transcription factors				
Ginkgolic acid	Ginkgo	SUMO2/3	p53	Guo et al. (2015)
		SUMO1	Smad4	Liu et al. (2020)
Astragaloside IV	*Astragalus membranaceus*	SUMO1	HIF-1α	Wang et al. (2021)
		SENP1	—	Liu et al. (2021)
		SUMO1	—	Wang et al. (2021a)
Resveratrol	Vitis L., Veratrum L., *Arachis*, Polygonum	SUMO1	β-catenin	Wang et al. (2020)
		SUMO1	COX-2	Cheng et al. (2018); Lin et al. (2011)
Puerarin	Radix Puerariae	SUMO2	ER/ERK	Zhao et al. (2021)
Gallic acid	gallnut, sumac, tea leaves, and oak bark	SENP1	HIF-1α	Taghvaei et al. (2021)
Ginsenoside Rg3	Panax ginseng	E3	NF-κB	Zou et al. (2018)
Modulating key mediators				
Ginkgolic acid	Ginkgo	SUMO1 and SUMO2/3	nephrin	Tossidou et al. (2014)

Astragaloside IV, isolated from *Astragalus membranaceus*, plays a key role in antioxidant, antifibrosis, antitumor and anti-diabetic effects (Zhang et al., 2020). Astragaloside IV improves angiogenesis in adverse hypoxic conditions through stabilizing the presence of HIF-1α protein via enhancing SUMO1 expression (Wang B. et al., 2021).

Additionally, natural products also play protective effects via SUMOylation in extrarenal tissue. Wnt/β-catenin signaling pathway plays a key role in promoting fibrosis (Li. S. S. et al., 2021). Resveratrol, a natural active antioxidant, alleviates inflammatory response and fibrosis by modulating SUMO1 through Wnt/β-catenin pathway in dextran sodium sulfate-induced inflammatory bowel disease mice (Wang et al., 2020). Resveratrol also induces SUMOylated cyclooxygenase (COX)-2 by SUMO1, thus enhancing the expression of pro-apoptotic genes via activating p53 in human prostate cancer LNCaP cells and human ovarian carcinoma (OVCAR-3) cells (Lin et al., 2011; Cheng et al., 2018). In heart failure mice, astragaloside IV reduces the levels of reactive oxygen species (ROS), and improves cardiac function by inhibiting the overexpression of SENP1 (Liu et al., 2021). Meanwhile, astragaloside IV promotes the proliferation and migration of vascular endothelial cells and antagonizes the adverse microenvironment of hypoxia and high glucose by enhancing SUMO1 expression in human umbilical vein endothelial cells (Wang B. S. et al., 2021). In cardiomyocytes, puerarin attenuates cellular inflammatory response and fibrosis through activating ER/ERK pathway by increasing the expression of SUMO2 (Zhao et al., 2021a). In oral squamous cell carcinoma cells, ginkgolic acid suppresses tumorigenicity and tumor progression through inhibition of the enhancement of SUMOylation of Smad4 (Liu et al., 2020). Ginsenoside Rg3, extracted from Panax ginseng, inhibits NF-κB signaling through increasing the phosphorylation of RanBP2 (E3 SUMO-protein ligase) in breast cancer cells (Zou et al., 2018).

Numerous studies have demonstrated that SENP1 could bind to HIF-1α for deSUMOylation (Bai et al., 2021; Taghvaei et al., 2021). Gallic acid plays an essential role in antioxidant, anti-inflammatory, antimutagenic and anticancer through reducing deSUMOylation via inhibiting SENP1 expression (Taghvaei et al., 2021). Natural products against renal fibrosis via regulating the SUMOylation of transcription factors are present in Figure 1.

### 4.2 Natural Products Against Renal Fibrosis via Regulating the SUMOylation of Key Mediators

Ginkgolic acid, extracted from Ginkgo biloba leaves and seed coat, have beneficial effects including anti-inflammatory (Li et al., 2018), antitumor (Zhao Q. et al., 2021), and anti-bacterial effects (Bhutta et al., 2021). Unfortunately, ginkgolic acid could aggravate kidney damage by enhancing apoptosis and downregulating the expression of nephrin. Ginkgolic acid enhances apoptosis through suppressing SUMOylation by SUMO2/3 and sensitizes renal tubular cells to apoptosis during cisplatin treatment of rat kidney proximal tubular cells (Guo et al., 2015). Additionally, ginkgolic acid reduces membrane expression of nephrin via suppressing SUMOylation by SUMO1 and SUMO2/3, leading to a significant proteinuria in mice (Tossidou et al., 2014). Natural products against renal fibrosis via regulating the SUMOylation of key mediators are present in Figure 2.

## 5 Discussion and Conclusion

In summary, the present study provides a systemic review of the role of SUMOylation in renal fibrosis, and the therapeutic effects of natural products through modulating transcription factors and key mediators. The pathological processes of renal fibrosis are regulated by complex signal pathways and factors, thus leading to a series of stress responses. The imbalance of protein SUMOylation is involved signal pathways and factors in renal fibrosis. SUMOylation significantly affects renal fibrosis through modulating transcription factors and key mediators, including Smads, HIF-1α, p53, NF-κB, podocyte apoptosis pathway, OAT3-mediated transfer channels, and Wnt/β-catenin pathway.

However, the effective treatment via SUMOylation remains a formidable challenge. The lack of effective treatment for renal fibrosis indicates that a deep understanding of the molecular mechanisms contributing to renal fibrosis remain urgent. Fortunately, researches of natural products in modulating SUMOylation have been carried out recently, which probably reveals the role of SUMOylation in the progression of renal fibrosis soon, including astragaloside IV, resveratrol, puerarin, gallic acid, and ginsenoside Rg3.

Additionally, the techniques that help to high-throughput screening of effective natural products targeting SUMOylation also facilitate to discover the candidate drug for treating renal fibrosis *via* modulating SUMOylation. It is worth noting that natural products mentioned above still needs more attention since the lack of underlying mechanism are far not enough for a candidate drug clinically. The detailed and in-depth basic research are urgent to investigate the underlying mechanisms of natural products against renal fibrosis, and the finding in reveal novel signaling pathways and treatment targets are necessary.
